# Altered Distant Synchronization of Background Network in Mild Cognitive Impairment during an Executive Function Task

**DOI:** 10.3389/fnbeh.2017.00174

**Published:** 2017-09-22

**Authors:** Pengyun Wang, Rui Li, Jing Yu, Zirui Huang, Zhixiong Yan, Ke Zhao, Juan Li

**Affiliations:** ^1^Center on Aging Psychology, CAS Key Laboratory of Mental Health, Institute of Psychology Beijing, China; ^2^Department of Psychology, University of Chinese Academy of Sciences Beijing, China; ^3^Faculty of Psychology, Southwest University Chongqing, China; ^4^Institute of Mental Health Research, University of Ottawa Ottawa, ON, Canada; ^5^School of Education Science, Guangxi Teachers Education University Nanning, China; ^6^State Key Laboratory of Brain and Cognitive Science, Institute of Biophysics, Chinese Academy of Sciences Beijing, China

**Keywords:** mild cognitive impairment, executive function, background network, degree of centrality, working memory

## Abstract

Few studies to date have investigated the background network in the cognitive state relying on executive function in mild cognitive impairment (MCI) patients. Using the index of degree of centrality (DC), we explored distant synchronization of background network in MCI during a hybrid delayed-match-to-sample task (DMST), which mainly relies on the working memory component of executive function. We observed significant interactions between group and cognitive state in the bilateral posterior cingulate cortex (PCC) and the ventral subregion of precuneus. For normal control (NC) group, the long distance functional connectivity (FC) of the PCC/precuneus with the other regions of the brain was higher in rest state than that working memory state. For MCI patients, however, this pattern altered. There was no significant difference between rest and working memory state. The similar pattern was observed in the other cluster located in the right angular gyrus. To examine whether abnormal DC in PCC/precuneus and angular gyrus partially resulted from the deficit of FC between these regions and the other parts in the whole brain, we conducted a seed-based correlation analysis with these regions as seeds. The results indicated that the FC between bilateral PCC/precuneus and the right inferior parietal lobule (IPL) increased from rest to working memory state for NC participants. For MCI patients, however, there was no significant change between rest and working memory state. The similar pattern was observed for the FC between right angular gyrus and right anterior insula. However, there was no difference between MCI and NC groups in global efficiency and modularity. It may indicate a lack of efficient reorganization from rest state to a working memory state in the brain network of MCI patients. The present study demonstrates the altered distant synchronization of background network in MCI during a task relying on executive function. The results provide a new perspective regarding the neural mechanisms of executive function deficits in MCI patients, and extend our understanding of brain patterns in task-evoked cognitive states.

## Introduction

The term mild cognitive impairment (MCI) is generally used to refer to a transitional zone between normal cognitive function and clinically diagnosed Alzheimer’s disease (AD; Winblad et al., 2004). Individuals with MCI display certain forms of cognitive dysfunction, but still maintain the intact ability to perform basic daily activities (Winblad et al., 2004).

Executive function is considered to be the mechanism that integrates the operations of various neural systems (McCabe et al., 2010; Funahashi and Andreau, 2013). Individuals with MCI show significant cognitive deficits in executive function compared to age-matched controls (Saunders and Summers, 2011; Clément et al., 2013). Generally, executive function is thought to include three components, mental set shifting, inhibition of prepotent responses and working memory (Garon et al., 2008). Working memory component in the framework of executive function refers to information updating and monitoring (Garon et al., 2008; McCabe et al., 2010). Many studies have been conducted to investigate working memory deficits in MCI patients (Klekociuk and Summers, 2014; Kirova et al., 2015). In terms of the corresponding neural mechanism, previous studies mainly investigated altered activation during working memory tasks (Bokde et al., 2010; Lou et al., 2015; Migo et al., 2015), which reflect the response of specific external stimuli. In recent years, several studies have investigated the background network during working memory task in MCI patients. For example, Lou et al. (2015) investigated the background network efficiency during the working memory state, and indicated that MCI patients showed increased background network efficiency to compensate the decreased activity and to maintain the working memory state (Lou et al., 2015).

In our previous study, we found altered local synchronization (indexed by regional homogeneity, ReHo) in MCI patients during working memory task relative to resting state (Wang et al., 2016). The index used in this study was the ReHo which employed Kendall’s coefficient of concordance to measure the coordination of activity between the voxel’s BOLD time series and those of its 26 nearest neighboring voxels to yield a voxel-wise ReHo map (Zang et al., 2004). Thus, it reflected a very local characteristic of the background network in MCI patients during working memory. However, whether the distant synchronization of the background network is changed in MCI patients during working memory functions is still unknown.

In graph theory, a complex system is modeled as a graph, which is defined as a set of nodes linked by edges. For a binary graph, degree of centrality (DC) is the number of edges connecting to a node. For a weighted graph, DC is defined as the sum of weights from edges connecting to a node, which is also sometimes referred to as the node connectivity strength (Zuo et al., 2012). This measure can be formalized as follows:

$$\text{DC}\left(i\right)\;=\;\sum_{j\;=\;1}^{N}w_{ij}$$

where *i* is the focal node, *j* represents all other nodes, *N* is the total number of nodes, *w* is the weighted adjacency matrix. *w_ij_* is greater than 0 if the node *i* is connected to node *j*, and the value represents the weight of the tie (for detail graph demonstration, see Opsahl et al., 2010). On a voxel-wise base, we distinguished local connectivity and distant connectivity using 14 mm as a demarcation point (within a voxel’s 14 mm neighborhood, or outside of its 14 mm neighborhood). A 14 mm radius was chosen following Sepulcre et al. (2010) as they observed stable estimates of distant connectivity for radius more than 10–14 mm. In this principle, the long DC (radius more than 14 mm) can be used as the index of distant (short and long) synchronization of the brain network (for detail, see Huang et al., 2016). Additionally, both positive and negative functional connectivity (FC; based on Pearson correlation coefficients) are reported here.

In the present study, using the DC index, we investigated distant background connectivity in MCI patients during working memory in the four conditions: correlations (positive vs. negative) by distance (local vs. distant). We hypothesized that distant connectivity in some regions would be impaired in MCI patients compared with normal healthy older adults during working memory functions.

## Materials and Methods

### Participants

In total, 17 MCI patients (age, 70.53 ± 4.54 years) and 16 healthy normal control (NC) elderly adult subjects (age, 68.56 ± 5.76 years) participated in this study. Participants were recruited from a community-based screening data pool in Beijing (healthy older adults, *n* = 865; MCI, *n* = 115; dementia, *n* = 21; Yu et al., 2012, 2014; Yin et al., 2015). MCI was diagnosed according to the diagnostic criteria for MCI (Petersen et al., 1999, 2001) and supplemented by scores from the Montreal Cognitive Assessment (MoCA; Nasreddine et al., 2005), Mini-Mental Status Examination (MMSE; Folstein et al., 1975) and Clinical Dementia Rating (CDR; Morris, 1993). For detail, please see the previous study (Wang et al., 2016). This study was approved by the research ethics committees of the Institute of Psychology, Chinese Academy of Science (H11036). Written informed consent was obtained from each participant.

### The Hybrid Delayed-Match-to-Sample Task (DMST)

Participants performed a modified hybrid delayed-match-to-sample task (DMST; Jiang et al., 2000; Lawson et al., 2007; Guo et al., 2008) in an functional magnetic resonance imaging (fMRI) scanner. The DMST is an executive function task and relies on the working memory component. The DMST was described in detail in our previous study (Wang et al., 2016). Briefly, during each trial, participants were asked to memorize two target objects which were presented side-by-side for 3500 ms. Then, the test objects (matching target objects or non-matching distractor objects) presented for 1000 ms. Participants needed to indicate whether a test object matched a target object by pressing corresponding button. The task requires participants to update and monitor the target and distractor pictures.

### Image Acquisition

Participants were scanned using a Siemens Trio 3.0 tesla scanner (Erlangen, Germany) at the Beijing MRI Center for Brain Research. During resting state scanning, participants were instructed to lie quietly with their eyes closed and not to think of anything in particular. For each participant, 200 resting state functional volumes were collected using the following parameters: TR = 2000 ms, flip angle = 90°, time echo (TE) = 30 ms, thickness = 3.0 mm, field of view (FOV) = 200 × 200 mm^2^, 33 axial slices, acquisition matrix = 64 × 64, gap = 0.6 mm, and in-plane resolution = 3.125 × 3.125. During working memory task scanning, the same parameters were used to collect 163 functional volumes for each run. Additionally, high-resolution 3-dimensional T1-weighted structural images were obtained for each participant using the following parameters: TR = 1900 ms, TE = 2.2 ms, flip angle = 9°, acquisition matrix = 256 × 256, 176 slices, and voxel size = 1 × 1 × 1 mm^3^.

### Behavioral Data Analysis

The response accuracy of working memory was calculated according to the hit rate (correct target detection) minus the false alarm rate (false report for distractors). Response times (RTs) were calculated as the mean response time for all test stimuli (targets and distractors). To consider the trade-off between accuracy and response time, working memory performance was further indexed using response accuracy divided by response time, which is the reciprocal of the “inverse efficiency score” previously reported (Kennett et al., 2001; Spence et al., 2001).

### Image Preprocessing

Functional MRI data, including both resting and task states, were preprocessed using the Statistical Parametric Mapping program (SPM8^1^) and the toolbox for Data Processing and Analysis of Brain Imaging (DPABI V1.3^2^; Yan and Zang, 2010). One-hundred and fifty-four volumes of both states were corrected for intra-volume acquisition time differences between slices using Sinc interpolation. Then, volumes were corrected for inter-volume geometrical displacement due to head motion using a six-parameter spatial transformation. All included participants had head motions less than 3 mm in any one direction during scanning. Coregistration, segmentation and writing normalization were conducted using unified segmentation of each participant’s T1 image. Normalized volumes were resampled to a voxel size of 3 × 3 × 3 mm^3^. Nuisance covariates, including head motion parameters, global mean signal, white matter signal and cerebral spinal fluid signal, were regressed out.

To make the resting and task state comparable, for the working memory run, an additional regressor of task conditions was included (e.g., targets and distracters present vs. absent trials; response vs. no response trials. See Jones et al. (2010) and Gordon et al. (2012) for the rationale for removing task-load effects). By this preprocessing step, resting and task data differed only in the subjects’ cognitive state. fMRI images were further spatially normalized to the Montreal Neurological Institute (MNI) echo planar imaging (EPI) template using an optimized 12-parameter affine transformation and nonlinear deformations. A high-pass filter (128 s cutoff period) was used to remove low-frequency confounds (see Lou et al., 2015). It was noted that as spatial smoothing may have artificially enhanced DC intensity that reduces reliability, just as the index of ReHo (Zuo et al., 2013), DC was calculated from an unsmoothed BOLD time series. Spatial smoothing was then performed using a 4-mm full-width at half-maximum (FWHM) Gaussian kernel.

### Whole Brain Degree of Centrality (DC)

The DC analysis was performed for each subject by GRETNA toolbox^3^ (Wang et al., 2015b). The principle was described in detail in our previous study (Huang et al., 2016). Briefly, the correlation between the time series of each voxel with every other voxel in the individual whole brain mask was computed. A binary, undirected adjacency matrix was then obtained by thresholding each correlation at *r* > 0.3 (following Huang et al., 2016). Based on the graph theory, DC was calculated at the individual level (Zuo et al., 2012). We computed DC by counting the number of functional connections (positive and negative correlations respectively) for each voxel to voxels inside (for local connectivity map) and outside (for distant connectivity map) of a 14 mm radius. Thus, we acquired four matrices for each group and cognitive state: positive local DC, positive distant DC, negative local DC and negative distant DC. In addition, normalized DC (DC-Z) indices were calculated by transforming DC to Z-scores based on the global mean of DC and standard deviation (SD) across voxels in the brain (Buckner et al., 2009; Zuo et al., 2012; Di Martino et al., 2013).

### Structural Image Analysis

Previous studies have reported gray matter (GM) atrophy in regions such as the medial temporal lobe, the frontal cortex and the parietal cortex of MCI patients (Singh et al., 2006; Karas et al., 2008). To control for the influence of GM atrophy on our DC analysis, a voxel-based morphometry (VBM) analysis was performed for structural images using DPABI to identify regions of GM atrophy. See the previous study for the detailed method and results (Wang et al., 2016). Briefly, MCI patients exhibited significant GM loss in several brain regions relative to NCs: the middle part of the medial frontal lobe, including parts of the cingulate gyrus, the limbic lobe and nearby white matter and the lateral frontal and parietal lobes. To analyze the effects of GM atrophy on the functional results, we performed the following repeated-measures analysis of variance (ANOVA) in which the GM intensity maps were entered as covariates.

### Between-Group Comparison of DC in Resting and Background Network of Working Memory States

To determine the interaction effects of group and cognitive state on DC, we performed a 2-way repeated-measures analysis of co-variance (ANCOVA) using SPM8, with group (MCI and NC) as a between-subject factor and cognitive state (resting and working memory) as a repeated-measure, controlling for age, gender, education, head motion and GM atrophy. *Post hoc* 2-sample *t*-tests were performed on clusters showing significant group × state interactions. The statistical threshold was set at *p* < 0.05 using the AlphaSim correction for multiple comparisons with a threshold of *p* < 0.01 at the voxel level. All coordinates are reported in the MNI format.

### Functional Connectivity Analysis

To examine the changes of FC from resting to task states in regions showing significant group × state interactions, we conducted a seed-based connectivity analysis with regions showing group × state interactions as seeds. First, for the background network during working memory task in each MCI or NC individual, voxel-wise FC maps to a given seed were computed as maps of temporal correlation coefficients between the BOLD time course of each voxel and the averaged BOLD time course across voxels in the seed region. FC maps from individual subjects were then transformed using Fisher’s Z transformation. Second, to determine the interaction effects of group and cognitive state on the FC maps, we performed 2-way repeated-measures ANOVA with group (MCI and NC) as a between-subject factor and cognitive state (resting and working memory) as a repeated-measure. *Post hoc* 2-sample *t*-tests were performed on clusters showing significant group × state interactions. The statistical threshold was set at *p* < 0.05 using the AlphaSim correction for multiple comparisons with a threshold of *p* < 0.01 at the voxel level.

### Correlation between State-Related Changes and Working Memory Performance

We calculated the Pearson correlation coefficient to explore the relationship between state-related changes of DC as well as corresponding FC and working memory performance (the quotient measured as response accuracy divided by response time) in regions showing significant interaction between groups and states. Bootstrap results were based on 1000 bootstrap samples, and 95% confidence interval were reported.

### Global Graph Theory Measures

To characterize the topological organization of networks, global efficiency and modularity were calculated on a voxel-level graph, using the GRETNA toolbox mentioned above. Global efficiency is biologically meaningful as it reflects how well the information propagates over a network (Rubinov and Sporns, 2010). Modularity measures the fraction of within-module edge weights in an actual network minus the expected value of this fraction in a network with the same community divisions, but the connections are randomly arranged between the nodes (Wang et al., 2015a).

## Results

### Alteration of DC

**Figure 1:**
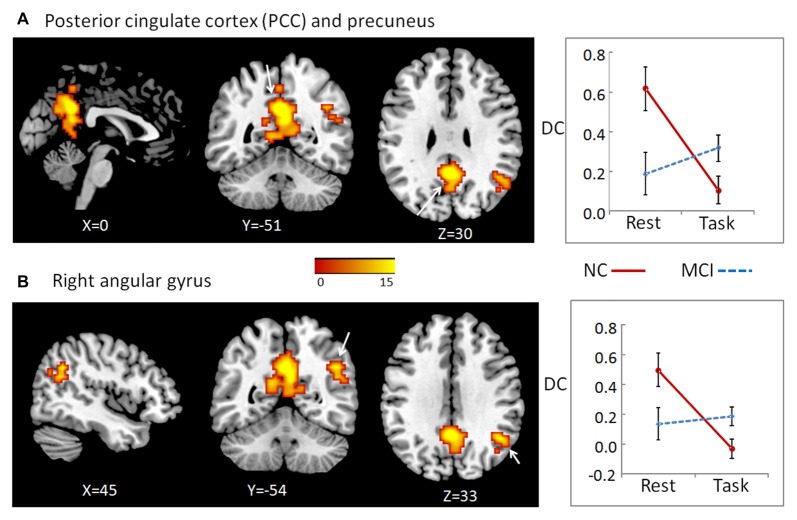
To determine the interaction effects of group and cognitive state on degree of centrality (DC), we performed a 2-way repeated-measures analysis of co-variance (ANCOVA), with group (MCI and NC) as a between-subject factor and cognitive state (resting and working memory) as a repeated-measure, controlling for age, gender, education, head motion and gray matter (GM) atrophy. We observed significant results only in positive long distance condition, which located in two clusters: the bilateral posterior cingulate cortex (PCC)/precuneus **(A)**, as well as right angular gyrus **(B)**. The color bar demonstrates *F* values. The corresponding graphs show the interaction patterns (right panels). Error bars depict standard error of the means (SEM). MCI, mild cognitive impairment; NC: normal control.

We did not find any stable or significant interactions between group and state in the positive local, negative distant, or negative local conditions. We observed significant results only in positive long distance condition located in two clusters. One was the bilateral precuneus (cluster size = 258) and posterior cingulate cortex (PCC, cluster size = 154), peak MNI coordinates: *x* = 0, *y* = −51, *z* = 30, and the other one was located in the right angular gyrus (cluster size = 67), peak MNI coordinates: *x* = 45, *y* = −54, *z* = 33 (Figure 1). For both clusters, further *post hoc t*-tests revealed that the value of DC decreased in the working memory state relative to the resting state in NCs, but did not significantly change in MCI patients (right panel of Figure 1 and Table 1).

**Table 1:** Regions showing significant interactions between group (mild cognitive impairment, MCI and normal control, NC) and cognitive state (resting and working memory).

Brain regions	NC (*n* = 16) Mean ± SD		MCI (*n* = 17) Mean ± SD		*F*	*p*
	Rest	Task	Rest	Task
DC of precuneus/PCC	0.617 ± 0.569	0.106 ± 0.296	0.190 ± 0.269	0.319 ± 0.258	15.22	<0.001
DC of right angular gyrus	0.498 ± 0.485	−0.031 ± 0.243	0.136 ± 0.406	0.186 ± 0.272	13.24	0.001
FC between precuneus/PCC and right IPL	0.006 ± 0.120	0.168 ± 0.108	0.057 ± 0.102	0.057 ± 0.085	12.24	0.001
FC between right angular gyrus and right anterior insula	−0.055 ± 0.105	0.212 ± 0.119	0.055 ± 0.138	0.078 ± 0.112	21.15	<0.001

*Notes: DC, degree of centrality; SD, Standard deviation; FC, functional connectivity; PCC, posterior cingulate cortex; IPL, inferior parietal lobule*.

The MCI patients showed significant deficit in working memory performance compared with NC, which was reported in our previous study (Wang et al., 2016). Furthermore, the state-related change (task minus resting) of DC in cluster precuneus and PCC correlated negatively with working memory performance in all participants (including both NC and MCI; *r* = −0.507, *p* = 0.003, bootstrap-based 95% confidence interval −0.693, −0.309), and in the MCI group alone (*r* = −0.502, *p* = 0.040, bootstrap-based 95% confidence interval −0.854, −0.195). For cluster right angular gyrus, the state-related change of DC correlated negatively with working memory performance in all participants including both NC and MCI (*r* = −0.390, *p* = 0.025, bootstrap-based 95% confidence interval −0.656, −0.118), but not in the MCI group alone (*r* = −0.089, *p* = 0.733, bootstrap-based 95% confidence interval −0.565, 0.363).

### Functional Connectivity of Regions Showing Group × State Interactions during Working Memory

**Figure 2:**
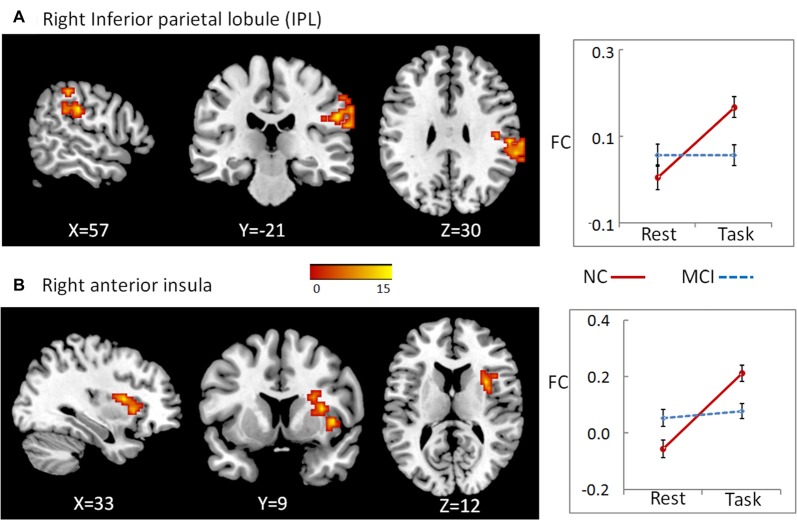
A seed-based connectivity analysis with regions showing group × state interactions as seeds was conducted to examine whether abnormal DC in these regions partially resulted from the deficit of FC between these regions and the other parts in the whole brain. For cluster bilateral PCC/precuneus, the significant region showing group × state interactions located in right inferior parietal lobule (IPL) **(A)**. For cluster right angular, we observed significant results in right anterior insula **(B)**. The color bar demonstrates *F* values. The corresponding graphs show the interaction patterns (right panels). Error bars depict SEM. FC, functional connectivity; MCI, mild cognitive impairment; NC, normal control.

To examine whether abnormal DC in these regions found as described above partially resulted from the deficit of FC between these regions and the other parts in the whole brain, we conducted a seed-based connectivity analysis with regions showing group × state interactions as seeds. Then, we compared the seed-based connectivity maps using 2-way repeated-measures ANOVA with group as a between-subject factor and cognitive state as a repeated-measure. For cluster bilateral PCC/precuneus, the significant region showing group × state interactions located in right inferior parietal lobule (IPL; **A**), cluster size = 87, peak MNI coordinates: *x* = 57, *y* = −24, *z* = 30 (Figure 2). For cluster right angular, we observed significant results in right anterior insula, cluster size = 56, peak MNI coordinates: *x* = 33, *y* = 9, *z* = 12 (Figure 2). For both clusters, further *post hoc* *t*-tests revealed that the value of FC was higher in the working memory state relative to the resting state in NCs, but did not significantly change in MCI patients (right panel of Figure 2 and Table 1).

Furthermore, the state-related change (task minus resting) of FC between right angular gurus and right anterior insula correlated positively with working memory performance in all participants (including both NC and MCI; *r* = 0.645, *p* < 0.001, bootstrap-based 95% confidence interval 0.425, 0.798), and in the MCI group alone (*r* = 0.579, *p* = 0.015, bootstrap-based 95% confidence interval 0.231, 0.829). The state-related change (task minus resting) of FC between cluster precuneus/PCC and IPL did not correlate with working memory performance in all participants or in MCI group alone, both *p*s > 0.05.

### Global Graph Theory Measures

**Figure 3:**
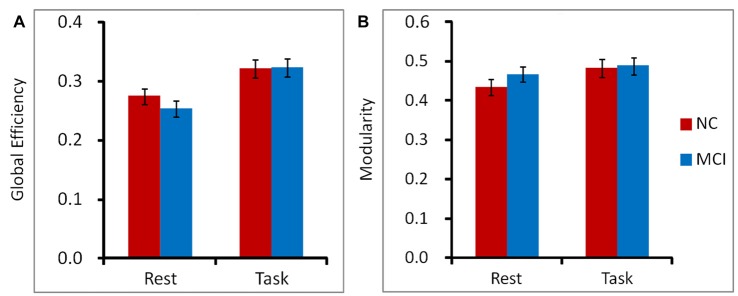
To characterize the topological organization of networks, global efficiency **(A)** and modularity **(B)** were calculated on a voxel-level graph. Participants demonstrated higher performance of global efficiency and modularity in working memory state than in rest state for both NC and MCI patients. The main effect of group and the interaction between group and cognitive state did not achieve significant for global efficiency or modularity. Error bars depict SEM.

We observed significant main effect of cognitive state in global efficiency, *F*_(1,31)_ = 8.66, *p* = 0.006, $\eta_{\text{p}}^{2}$ = 0.22. Participants demonstrated higher performance of global efficiency in working memory state than in rest state for both NC and MCI patients. The main effect of group and the interaction between group and cognitive state did not achieve significance, both *p*s > 0.05. The similar pattern was observed in modularity. Participants showed higher performance of modularity in working memory state than in rest state for both NC and MCI patients, *F*_(1,31)_ = 16.47, *p* < 0.001, $\eta_{\text{p}}^{2}$ = 0.35. There was no significant main effect of group or interaction between group and cognitive state, both *p*s > 0.05 (Figure 3).

## Discussion

The present study demonstrates the altered distant synchronization of background network in MCI during working memory task. We observed significant interactions between group and state in the bilateral PCC and the ventral subregion of precuneus. Specially, the results indicated that, for the NC group, the distant FC of the PCC/precuneus with the other regions of the brain was higher during rest state than that during working memory state. For MCI patients, this pattern was not evident. There was no significant difference between rest and working memory state. A similar pattern was observed in the other cluster located in the right angular gyrus. To examine whether abnormal DC in these regions found as described above partially resulted from the deficit of FC between these regions and the other parts in the whole brain, we conducted a seed-based connectivity analysis with these regions as seeds. The results indicated that the FC between bilateral PCC/precuneus and the right IPL increased from rest to working memory state for NC participants. For MCI patients, however, there was no significant change between rest and working memory state. The similar pattern was observed for the FC between right angular gyrus and right anterior insula.

PCC is the key structure of the default mode network (DMN; Menon, 2011; Patel et al., 2015). The ventral subregion of precuneus (next to PCC) is also wildly accepted as part of the DMN (Zhang and Li, 2012). Some studies have argued that the ventral subregion of precuneus is deactivated during successful memory encoding (Daselaar et al., 2004; Vannini et al., 2011). In addition other research has shown a tendency for negative correlations between the activations of this region and the task performance (Rami et al., 2012). Furthermore, the posterior IPL is also a part of the DMN (Andrews-Hanna et al., 2010). The results of the present study demonstrated that as a key structure of the DMN, the DC in region of PCC/precuneus in the brain network was higher in rest state than in task state. However, when normal participants transferred from the rest state to a working memory state, the DMN was no longer the key structure for the current cognitive task. Therefore, the DC in PCC/precuneus was lower in task state than in rest state. Nonetheless, the FC between PCC/precuneus and IPL was higher during the working memory state than the rest state. This pattern may demonstrate that although the activity of the regions belong to DMN decreased from rest to task state, the inner connectivity within the DMN increased, because the regions of DMN had to cooperate to be inhibited to complete the current cognitive task. Compared with NC, these patterns were not observed in MCI patients, which may result in the working memory deficit in MCI patients. It should be noted that the state-related alteration (task minus resting) of FC between cluster PCC/precuneus and IPL is not correlated with working memory performance in NC or in MCI group. This may indicate that the state-related alteration of FC between these two regions changed qualitatively from NC to MCI patients.

As a part of the default mood network, it is striking to see the high similarity in task-free deactivation in angular gyrus (Menon, 2011; Seghier, 2013). Some studies have reported that the deactivation of angular gyrus during encoding is beneficial for the memory performance (Daselaar et al., 2009; Uncapher and Wagner, 2009). Nevertheless, there were also opposite findings suggesting that the left angular gyrus activity was greater during successful vs. unsuccessful episodic encoding (Maillet and Rajah, 2014). Elman et al. (2013) demonstrated the dynamic changes in angular gyrus during encoding. The angular gyrus activity decreased when the stimulus initially presented in the memory task (participants were asked to make a perceptual judgment). Then, the stimulus disappeared. Several seconds later, when the participants were asked to remember the stimulus presented previously for a later memory test (an elaborative representational encoding process), the angular gyrus activity increased. Furthermore, as reviewed by Seghier (2013), angular gyrus is a cross-modal integrative hub. Specifically, it is an interface between the converging bottom-up multisensory inputs and the top-down predictions. Angular gyrus processes the information from insula and prefrontal regions. The anterior insula and the dorsal anterior cingulate cortex are the key structures of the salience network, which is involved in detecting, integrating and filtering relevant interoceptive, autonomic and emotional information (Seeley et al., 2007; Menon, 2011). These functions are important to complete the current working memory task.

As described in the introduction, DC is defined as the sum of weights from edges connecting to a node, which is also referred to as the node connectivity strength (Zuo et al., 2012). Thus, DC can be used to represent the importance of a brain region throughout the brain network. The results of the present study demonstrated that compared to MCI patients, the DC of right angular gyrus in NC participants was lower during working memory state compared with rest state, but the connectivity between right angular gyrus and right anterior insula increased. Therefore, the results may indicate that compared to MCI patients, although the importance of right angular gyrus (represented using DC) in the network in NC participants decreased from rest to working memory state, the FC of angular gyrus was more converging on the right insula which facilitated the current cognitive task. The positive correlation between FC and the working memory performance confirmed this relationship further. Relative to NC, MCI patients lacked corresponding changes (as described above) from rest to working memory state, which may reflect their working memory deficit.

We observed significant main effect of cognitive state in global efficiency and modularity. Participants showed higher global efficiency and modularity in working memory state than in rest state. However, there was no difference between MCI and NC groups. Considering the changes described above (the PCC/precuneus, angular gyrus and the FC between PCC/precuneus and IPL, as well as the FC between angular gyrus and anterior insula), it may indicate that the brain network of MCI patients did not show large scale FC changes, on the contrary, it demonstrated a lack of efficient reorganization from rest state to a working memory state. Of note, we observed these different reorganization patterns between NC and MCI patients only in the long distant positive FC condition. This phenomenon was not found in negative FC or in the positive FC in physically short distances of the brain (i.e., local networks).

It should be noted that our study accounted for the possible influence of GM structural atrophy on our findings. To limit the effects of GM atrophy on the functional results, during the DC analysis, we performed the repeated-measures ANOVA in which the GM intensity maps were entered as covariates. Actually, the VBM analysis showed that MCI patients demonstrated GM atrophy in the middle part of the medial frontal lobe and in the lateral parts of frontal and parietal lobes (Wang et al., 2016), however, all these regions did not overlap with regions showing interactions between group and state.

As mentioned in our previous study (Wang et al., 2016), the limitations of the present study were largely related to the small number of MCI patients and the uncontrolled subtypes and severity of cognitive impairment in MCI patients. In addition, the sub-processes of the working memory components (such as encoding, maintain and retrieval) also need to be considered in the further studies.

Our previous study found the altered local synchronization (indexed by ReHo) in MCI patients during working memory task relative to resting state (Wang et al., 2016). The current study expands our previous work by finding the altered distant synchronization of the background network in MCI patients during working memory, which may also serve as a parameter for disease diagnosis and progression monitoring in MCI patients. To our knowledge, this is the first study to examine alterations of distant FC of the background network during working memory in MCI patients. Similar to our previous study (Wang et al., 2016), the present study provides a new perspective regarding the neural mechanisms of working memory deficits in MCI patients, and extends our knowledge of altered brain patterns in resting and task-evoked states.

## Author Contributions

PW conceived the idea, designed the study, analyzed and interpreted data, and drafted part of the manuscript. RL, ZH, KZ and ZY assisted with the analysis and interpretation of data. JY conducted the experiment. JL conceived the idea, designed the study and participated in the writing and revision of the manuscript.

## Conflict of Interest Statement

The authors declare that the research was conducted in the absence of any commercial or financial relationships that could be construed as a potential conflict of interest.

## Footnotes

^1^http://www.fil.ion.ucl.ac.uk/spm

^2^http://rfmri.org/dpabi

^3^http://www.nitrc.org/projects/gretna/
